# Perioperative quantitative coronary analysis and ultrasonographic graft assessments for the right coronary artery bypass grafting

**DOI:** 10.1186/1749-8090-10-S1-A200

**Published:** 2015-12-16

**Authors:** Shinya Takahashi, Keijiro Katayama, Taijiro Sueda

**Affiliations:** 1 Department of Cardiovascular Surgery, Hiroshima University Hospital, Hiroshima, 7348551, Japan

## Background/Introduction

Preoperative quantitative assessment of coronary angiography (CAG) and real-time graft size and graft flow assessment using epigraftic ultrasonography in the operative field have been performed to ascertain the patency in the right coronary artery bypass grafting (CABG).

## Aims/Objectives

This study aims to evaluate the efficacy of the quantitative coronary artery and graft assessment to increase the patency of the right CABG.

## Method

CABG was performed in 200 patients from January 2010 to December 2014. Ninety-three patients underwent all of preoperative CAG, real-time graft assessment and postoperative CAG with a total of 93 grafts anastomosed to the right coronary artery. Severity of coronary artery stenosis was evaluated by CAG. The size of target right coronary artery was measured by ultrasonography in the operative field. Parameters about graft flow were obtained from flow velocity curve in all the graft. The grafts were divided into two groups: patent grafts (Group A, n = 86) and failing grafts (Group B, n = 7). All factors were compared in these two groups and evaluated by logistic analysis and receiver operating characteristic (ROC) curve analysis.

## Results

The overall patency as measured by postoperative CAG was 94.6% (88/93). There were 7 failing grafts including 5 occlusion and 2 competitive slow flow. Logistic regression analysis revealed that the percentage of graft size measured by ultrasonography divided by the size of the right coronary artery (graft-RCA size mismatch) and the pulsatility index (PI) were independent predictors of early graft failure (graft-RCA size mismatch, Odds ratio [OR], 2.37, 95% confidence interval [95%CI], 1.94-289.0, p = 0.0132; PI, OR, 1.51, 95%CI, 1.16-1.96, p = 0.0023, area under the curve [AUC], 0.981). ROC curve analysis revealed that graft-RCA size mismatch > 2.08 and PI > 4.55 were predictors of graft failure.

## Discussion/Conclusion

In this series, epigraftic ultrasonography depicted graft flow clearly. Combination of preoperative quantitative coronary artery assessment and real time graft assessment was essential to predict graft failure during the CABG. This technique may increase the patency of the right coronary artery bypass grafting.

